# The Benefits of Spiritual Diaries: A Mixed-Method Study in Korea

**DOI:** 10.1007/s10943-021-01277-8

**Published:** 2021-05-12

**Authors:** Suk-Sun Kim, Yeounsoo Kim-Godwin, Minji Gil, DaEun Kim, Yeon Kum Cheon

**Affiliations:** 1 College of Nursing, Ewha Womans University, 52, Ewhayeodae-gil, Seodaemun-gu, Seoul, 03760 Korea; 2 School of Nursing, College of Health and Human Services, University of North Carolina Wilmington, 601 S. College Road, Wilmington, NC 28403 USA

**Keywords:** Spiritual diary, Spiritual growth, Psychological well–being, Mixed-method study

## Abstract

This mixed study examined the benefits of spiritual diaries in Korea. Quantitatively, differences in spiritual growth and psychological well-being were examined in relation to the frequency of writing spiritual diaries among 385 participating adults. The group who wrote spiritual diaries ‘5–7 times a week’ had significantly higher scores relating to spiritual growth and psychological wellbeing than other groups across the outcome variables. Qualitatively, the study also explored the benefits of writing spiritual diaries among 37 adults. Three major themes using four focus group interviews, were identified: (a) the acquisition of godly habits, (b) a closer walk with Jesus, and (c) the fullness of God’s presence. These findings are particularly important for healthcare providers who want to facilitate patient self-care.

## Introduction

Spirituality is an important resource in physical and psychological well-being. Numerous researchers reported that people who have higher spirituality show better mental health and adapt more quickly to health problems, compared to those who have lower spirituality (Doolittle, Justice, & Fiellin, 2018; Park & Slattery, 2013; Salsman et al., 2015). However, few studies have evaluated the effectiveness of various spiritual interventions on mental health. A systematic review found that the use of prayer in clinical practice reduced the anxiety of mothers of children with cancer, improved physical functioning of patients, and increased the in vitro implantation rate for successful pregnancies (Simão, Caldeira, & de Carvalho, 2016). For holistic healthcare, it is crucial to provide spiritual activities as self-care practices because such activities can enhance spiritual well-being and quality of life and alleviate depression (White, 2016).

Keeping a spiritual diary on the website can be an effective online religious activity that integrates spiritual beliefs with expressive writing. Using an Internet website and application for writing spiritual diaries and then sharing them with church members allows people to communicate with God and others in cyberspace (Yoo & Noh, 2018). It records what God has done in one’s life and reflects upon one’s walk with Jesus in everyday moments. It is not only a record of God’s involvement in one’s life, but also the daily experience of God’s companionship (Yoo, 2017). From the Old Testament Psalms and Ecclesiastes to Augustine's confessions, John Wesley, and Frank Rubach, a spiritual diary was used as a method of spiritual discipline to record the spiritual journey of living one's life in the presence of God at every moment (Park, 2010). In Korea, Pastor Kisung Yoo initiated a movement of writing spiritual diaries at his church. Since then, it has been introduced to other Korean churches through the website (http://wjsm.kr/) and the mobile application.

Moreover, ‘*Spiritual Journal,’* a spiritual diary guide, is spreading across the world (English, Chinese, Taiwanese, Japanese, Russian, and Indonesian) (Yoo, 2017). Currently, around 100,000 people from 1268 churches in 173 countries have participated in writing spiritual diaries (With Jesus Ministry, 2020). The format of a spiritual diary is varied: a general diary format, guided format, confession format, quiet time (QT) journal, and gratitude writing (Kim, 2017; Wesley, 1996; Yoo, 2017). A general diary format records one's life in the presence of God at every moment. A guided format helps the beginner to write spiritual diaries by answering four guideline questions (i.e., When do you think of Jesus in a day?; How do you feel when you think of Jesus in a certain situation and what happens or changes when you acknowledge him?; What special things can you remember during the day and how did Jesus work at those moments?; What has happened when you obeyed [or disobeyed] the promptings of Holy Spirit [Jesus] given to you?) (Yoo, 2017). Confessional writing includes looking back on a day and repenting of sin. The QT journal involves reading the Bible’s passage carefully and writing down thoughts and responses related to the passage (e.g., What can you learn about God?; Are you learning from God’s teaching?; and How to apply this lesson into the life?). Lastly, gratitude writing records what one is grateful for during a day (Schnitker & Richardson, 2019).

Spiritual diaries can help participants consistently look to Jesus, build a loving relationship with God and others, progressively release fear and anxiety, increasingly maintain a holy life, and live a life beyond oneself through the identity of being a people called by God (Yoo, 2017). However, there has been a paucity of studies exploring the benefits of writing spiritual diaries for spiritual growth, as well as mental and physical health. Park (2010) assigned 40 college students the task of writing three-week spiritual diaries, asking them to report their overall experience in terms of positive and negative impacts. The students reported that they could see life meaningfully through the spiritual diary, experiencing spiritual and psychological health effects, increased gratitude and mental relaxation, and reduced stress. However, the results of this research were difficult to generalize because it was a case study with a small sample size. Therefore, this study explored the benefits of spiritual diaries on spiritual growth and psychological well-being quantitatively and qualitatively. It will contribute to the development of evidence-based spiritual interventions for health and well-being.

## Method

### Study Design

This study used the explanatory sequential design (J. W. Creswell & Creswell, 2017), which is a three-phase mixed method to explain the benefits of spiritual diaries. First, we conducted quantitative research to examine differences in spiritual growth and psychological well-being in relation to the frequency of writing spiritual diaries among 385 participating adults. The quantitative results in the preliminary study were reported in Korean (Kim, Cheon, Choi, & Gil, 2018). The findings indicated that participants who wrote spiritual diaries 5–7 times a week had higher spiritual growth and psychological well–being than those who wrote spiritual diaries less frequently.

To make an interpretation and elaborate on these results, qualitative research was implemented in the second phase. The qualitative results explored more in-depth the experiences among adults who previously participated in the quantitative study. Lastly, the integration phase combined and interpreted quantitative and qualitative findings. These integrated findings reported here focus on exploring why writing spiritual diaries daily promotes spiritual growth and psychological well-being, using interviewees who wrote spiritual diaries daily.

### Participants and Data Collection

The Institutional Review Board of Ewha Womans University approved the study. Participants provided informed consent prior to data collection. Data were collected from June 1, 2017, to April 30, 2018. A total of 385 adults who wrote spiritual diaries at a church in the Republic of Korea completed the survey. The inclusion criteria were: (a) being over the age of 19 and (b) having experience writing spiritual diaries more than once via the mobile application or website. However, those who have difficulties with communications (reading, understanding, and writing) were excluded.

For the focus group interviews (FGI), participants were recruited based on their response to the survey questions, their frequency of writing spiritual diaries, and a willingness to participate. Six homogeneous focus groups were composed based on their frequency of writing spiritual diaries and their overall church duties. Each group consisted of 9 to 11 people, and we interviewed them for 60 to 120 min with three broad, open-ended questions. In this mixed research, we used only 37 participants in four FGI who wrote a spiritual diary daily for the qualitative analysis.

### Measures

#### General Characteristics

The questionnaire included gender, age, education, marital status, years of religious life, and questions relating to spiritual diary characteristics. In particular, the frequency of writing a spiritual diary was measured on a 7-point scale, ranged from ‘haven’t written at all’ to ‘write daily.’ For statistical analysis, the 7-point scale was re-categorized into three groups: ‘5–7 times a week,’ ‘1–4 times a week,’ and ‘less than once a month.’

#### Spiritual Growth

Spiritual growth was measured using the Spiritual Perspective Scale (SPS: Reed, 1987) and the Self-Transcendence Scale (STS: Reed, 1986), which captured a broad notion of spiritual growth including spiritual perspective and multidimensional transcendence. The Cronbach alpha of the Korean version of SPS-K (*α*=.97) and STS-K (*α*=.85) supported that both had good internal consistency. A moderate association (*r*=.45, *p*<.01) between the SPS-K and the STS-K supported the convergent validity of the instruments (Kim et al., 2012).

The Korean version of the SPS (Kim et al., 2012) measures the extent of spiritual perspective and engagement in spiritual activities in a person’s life. The 10-items of the SPS are rated on a 6-point Likert scale from ‘not at all’ to ‘very much,’ with higher scores reflecting a greater level of spiritual perspective. The Cronbach alpha in this study was 0.84.

The Korean version of the STS (Kim et al., 2012) is a 15-item questionnaire that assesses self-transcendence as an expansion of personal boundaries in multiple ways: inwardly through introspective activities, outwardly through involvement with others, transpersonally by reaching toward spiritual dimensions, and temporally by living in the present or holding perspectives on the past and future that enhance the present. Each item is rated on a 4-point Likert scale from ‘not at all’ to ‘very much,’ with higher scores presenting a greater self-transcendence. The Cronbach alpha in this study was. 82.

#### Psychological Well-Being

Psychological well-being was measured using the Satisfaction with Life Scale (SWLS; Diener et al., 1985) and the Center for the Epidemiological Studies-Depression Scale (CES-D: Radloff, 1977), which captured both cognitive and emotional aspects of psychological well-being.

The Korean version of the SWLS (W. Kim & Kim, 1997) is a 5-item scale that measures overall life satisfaction. Each item is rated on 7-point Likert scale with higher scores reflecting a higher level of satisfaction in life. The alpha coefficients (*α* = 0.86) showed good internal consistency (W. Kim & Kim, 1997). Also, the convergent validity was supported by the positive correlation between the SWLS and both the Life Satisfaction Index (*r* = 0.46, *p* < 0.001) (Diener et al., 1985) and Life Orientation Test (*r* = 0.39, *p* < 0.001) (W. Kim & Kim, 1997). The Cronbach alpha in this study was 0.92.

The Korean version of the CES-D (Cho & Kim, 1993) is a 20-item scale that measures the symptoms of depression. Each item is rated on a 4-point Likert scale with higher scores reflecting a greater experience of depressive symptoms during the previous week. The alpha coefficients (*α* = 0.90) and the test–retest coefficient (*α* = 0.68) indicated acceptable reliability. A high association (*r* = 0.82, *p* < 0.001) with the Beck Depression Inventory (Beck et al., 1961) supported the convergent validity (Cho & Kim, 1993). The Cronbach alpha in this study was 0.92.

#### Focus Group Interview Questions

Three broad, open-ended questions regarding the experiences of writing spiritual diaries daily were asked:

- (1) What motivates you to write a spiritual diary every day?
- (2) What do you experience while writing spiritual diaries daily?
- (3) Are there any differences in your life after writing daily spiritual diaries?

These questions prompted discussion about their experience of writing spiritual diaries, and subsequent questions were used to trigger recollections and clarify participants’ responses.

## Data Analysis

Data were analyzed using the SPSS Statistics 23.0 for Windows, and all statistical significance levels were adopted at 0.05. Descriptive statistics were calculated for all variables of interest. Analysis of variances (ANOVA) were used to examine the differences in spiritual growth and psychological well-being among three groups: ‘5–7 times a week,’ ‘1–4 times a week,’ and ‘less than once a month.’ For the post hoc test, Scheffe Test and Dunnett T3 were used.

For qualitative data analysis, the content analysis method (Bengtsson, 2016) was used to analyze responses to the three open-ended questions. This method systematically analyzed the focus groups’ interview data and identified what participants said and the meaning behind what they spoke. First, three researchers read audio-taped and transcribed interviews, coded the data, and discussed and resolved any discrepancies in coding. The researchers identified 15 categories and then built a list of themes and subthemes through collaboration. Second, they merged the 15 categories into three major themes with six subthemes. Finally, a table was created to organize the identified themes, subthemes, and categories.

## Results

### Demographics and Characteristics Related to Spiritual Diaries

Descriptive statistics for demographics are presented in Table 1. The 385 samples consisted of 143 men (37.1%) and 242 women (62.9%). The age of the subjects ranged from 20 to 77 years, with a mean age of 47.69 ± 10.16. Also, the mean years of religious life were 31.65 ± 14.52.

**Table 1:** Demographic and Spiritual Diary-related Characteristics (*N* = 385)

Demographic characteristics	*n*	%
*Gender*		
Male	143	37.1
Female	242	62.9
*Age*	47.69 ± 10.16	
Under 39 years old	76	19.7
40 ~ 49 years old	122	31.7
50 ~ 59 years old	117	30.4
Over 60 years old	49	12.7
Missing data	21	5.5
*Education*		
High school graduation	86	22.3
Associate degree	55	14.3
Bachelor’s degree	145	37.7
Master’s and Doctoral degree	99	25.7
*Marriage status*		
Married	304	79.0
Non-marriage	79	20.5
Missing data	2	0.5
*Years of religious life*	31.65 ± 14.52	
Under 20 years	94	24.4
21 ~ 39 years	159	41.3
Over 40 years	126	32.7
Missing data	6	1.6

Spiritual diary-related characteristics		
*Frequency of writing spiritual diary*		
5 ~ 7 times a week	188	48.8
1 ~ 4 times a week	172	44.7
Less than once a month	25	6.5
Writing format*		
Typical diary format	145	37.7
Quiet time and sermon	115	29.9
Form of repentance	115	29.9
Spiritual diary guide writing form	91	23.6
Thanks diary	88	22.9
Motivation to start spiritual diary*		
Pastor's recommendation	227	59.0
Spontaneous occasion	102	26.5
Friends or neighbors’ recommendation	30	7.8
By the surrounding environment	18	4.7
Family’s recommendation	7	1.8
Reason of writing spiritual diary*		
To live with God	280	72.7
To check the spiritual life of the day	97	25.2
For a change of life	45	11.7
For the record of life	30	7.8
Obstacles in writing a spiritual diary*		
Insufficient time	196	50.9
Burden of diary release	115	29.9
Difficulty in writing	100	26.0
Absence of diary material	49	12.7
Benefits of spiritual diary on life*		
Growth of faith	336	87.3
Peace of mind	266	69.1
Change in relationships with people	263	68.3
Overcoming the problem of sin	262	68.1
Change in family life	230	59.7
Change in social attitude	230	59.7
A change in personality	211	54.8

^*^ Multiple responses

According to the frequency of writing spiritual diaries: 48.8% of the participants wrote ‘5–7 times a week’; 44.7% wrote ‘1–4 times a week’; 6.5% wrote ‘less than once a month.’ The most common reasons for writing spiritual diaries were ‘to live with God’ (72.7%), ‘to evaluate my spiritual life for the day’ (25.2%), ‘to make changes in my life’ (11.7%), and ‘in order to leave a record’ (7.8%). The participants could choose more than one answer related to spiritual diary characteristics, except for the frequency of spiritual diary writing (Table 1).

The 37 focus group participants consisted of 16 men (43.2%) and 21 women (56.8%). The ages ranged from 35 to 66 years, with a mean of 51.64 ± 9.03. The mean years of religious life were 37.32 ± 13.83. Duties in the church were as follows: ‘Senior Deacon’ (37.8%), ‘Deacon’ (24.3%), ‘Pastor’ (21.6%), ‘Elder’ (8.2%), ‘Pastor’s wife’ (5.4%), and ‘Church member’ (2.7%). The reasons for writing and benefits of the spiritual diaries were similar for both the focus group members (*n* = 37) and total subjects (*N* = 385).

### Quantitative Results: Group Differences in Spiritual Growth and Psychological Well-being

Table 2 illustrates the results of ANOVA and the post hoc tests with the means and standard deviations of all study variables by three groups: ‘5–7 times a week’ (Group A), ‘1–4 times a week’ (Group B), and ‘less than once a month’ (Group C). There were statistically significant differences in spiritual growth (SPS (*F* = 9.10, *p* < 0.001), STS (*F* = 8.33, *p* = 0.001)) and psychological well-being (SWLS (*F* = 8.16, *p* < 0.001), and CES-D (*F* = 7.00, *p* = 0.001)) among three groups. Also, the findings of the post hoc tests indicated that participants who wrote spiritual diaries ‘5–7 times a week’ (Group A) had significantly higher SPS, STS, and SWLS scores than other groups (Groups B and C). However, there were no statistically significant differences in CES-D between the groups.

**Table 2:** Group Differences in Spiritual Growth and Psychological Well-being

Variables		5–7 times a week (Group A)		1–4 times a week (Group B)		Less than once a month (Group C)		F(*p*)	Scheffe/Dunnett T3
		Mean	SD	Mean	SD	Mean	SD		
Spiritual growth	SPS	56.89	3.52	55.85	3.99	53.68	4.98	9.10 (< .001) ^***^	b, c < a ^†^
	STS	50.91	5.20	49.22	5.13	47.20	5.78	8.33 (.001) ^***^	b, c < a
Psychological well-being	SWLS	24.86	7.39	22.92	6.84	9.32	7.34	8.16 (< .001) ^***^	b, c < a
	CES-D	7.20	7.87	9.28	9.42	13.88	13.56	7.00 (.001)^***^	

^*^p < .05,^**^p < .01,^***^p < .001, post hoc: Scheffe, Dunnett T3^†^

*SPS* Spiritual Perspective Scale, *STS* Self-Transcendence Scale, *SWLS* Satisfaction with Life Scale, *CES-D* Center for Epidemiological Studies Depression

### Qualitative Findings: The Benefits of Spiritual Diaries

The 37 participants in the four FGI shared their rich experiences from writing spiritual diaries daily. Three major themes with six categories were identified: (a) the acquisition of godly habits, (b) a closer walk with Jesus, and (c) the fullness of God’s presence. While they formed a godly habit of writing spiritual diaries daily through spiritual disciplines and obedience, they moved toward a more intimate walk with God and could be assured of His constant presence.

### The Acquisition of Godly Habits

The theme of ‘the acquisition of godly habits’ was derived from two categories: spiritual disciplines and obedience. Participants utilized the spiritual diary as a tool of spiritual discipline, training themselves to walk with God in each and every moment. A participant explained that the spiritual diary is a collection of spiritual disciplines including reading the Bible passages, prayer, repentance, gratitude, and experiences with the presence of God:

I usually pray, have quiet time, and read the Bible, but I can’t remember God and can’t stay connected to God when my life swirls around me at a rapid pace. After I have written spiritual diaries, I am looking to Jesus and accompanying God in prayer, QT, and even when I am not reading the Bible. From the beginning of the day in the morning until the end of the night, I think that it is a spiritual diary to capture and record all other forms of spiritual discipline, while keeping the whole life walking with God (King, M/45).

The participants also reported that obedience in daily spiritual diary writing helped to form this godly habit:

In the Bible (Genesis 5:24), Enoch was called a man who walked with God, so I started writing every day because I wanted to walk with God like Enoch. I tried to spend time praying and talking with Him throughout the day. I think that writing daily SDs is an opportunity to obey and follow God to have an intimate relationship with Him (John, M/46).

The daily obedience in writing spiritual diaries is very important. If you don't write a day's diary, you're more likely not to write the next day, and if you miss two days, you won't write for a week. Daily habits began with the practice of spiritual diary discipleship. From then on, I tried to at least write a single line instead of missing even one day. For me, writing a spiritual diary every day seems to be a complete part of my life, just like I have to brush my teeth and take a shower before going to bed every day (Matthew, M/47).

Participants mentioned that writing a spiritual diary every day was not easy. Despite the various difficulties of writing spiritual diaries, participants considered daily spiritual diary keeping as an act of spiritual discipline and obedience to get closer to God. As a result, participants built godly habits of writing spiritual diaries daily and walking with God closely.

### A Closer Walk with Jesus

The theme of ‘a closer walk with Jesus’ was derived from two categories: enjoying an intimate relationship and focusing on God’s perspective through a better understanding of Jesus's life, death, and resurrection. Participants experienced an intimate relationship with God through Jesus by recording how God was working in their lives. Participants reported that while they tried to fix their eyes on Jesus throughout the day and journal daily, they could enjoy fellowship with God and successfully grasp His presence:

As I obey and write, I have more time to seek God and pray every day than ever before. As a result, the relationship with God becomes intimate, and the relationship with God naturally is expressed in a diary (Job, M/62).

When I write a spiritual diary, I record intimacy and my walk with God by daily obedience... If I don't write it every day, I will live with my thoughts and will [against the Spirit]. However, when I write every day, I can feel the presence of God all day long, so it helps me to have fellowship with God and develop spiritual growth (Esther, F/51).

Participants also mentioned that keeping a spiritual diary allowed them to promote a sense of connection to God. The more they drew close to God, the more they aligned their perspectives to God’s and changed their lives:

While I wrote in my diary, I revealed my heart to God. Responding to God's voice about the day's events and writing a diary made me think from God's perspective.

Misunderstandings of other people disappeared. I became less critical of myself. When I looked at the Lord with all my heart and wrote a diary, I set my mind and heart to seek God’s presence (Rahab, F/44).

Whenever I spend time writing a spiritual diary, I am able to reflect on my life and look towards God for guidance. If there is a problem to be resolved in my relationship with someone, it is solved by writing a diary. Solving the problems of the day by spiritual diary is self-examination and allowed to remove impurities every day. Writing an SD is a method of self-examination that removes the impurities of the day and helps uncover solutions for the problems of the day. So the next day, I can be free from the influence of the problem, and I think I'm increasingly living in awe of God and focusing on God's perspective (Joshua, M/38).

Participants stated that a spiritual diary is a tool for practicing companionship with the Lord. While they fixed their eyes on Jesus in every moment by using daily spiritual diaries, they were able to respond to what God is saying to them and think about what God has done. They also reinterpreted their life from God's perspective and experienced living with the presence of Jesus. [Through the belief in God as a Trinity, participants used the names God, Jesus, and LORD interchangeably].

### The Fullness of God’s Presence

The theme ‘the fullness of God’s presence’ was derived from two categories: repentance and transformation. Participants mentioned that daily spiritual diary writing helped them to reflect on what they had done and how well they walked with God in the daily life. So, they became sensitive to wrongdoing and were able to repent sins promptly:

As I looked back at how I walked with God throughout the day, as I wrote my spiritual diary, I repented and confessed my sins. After that, I didn't want to do the same thing. By contrast, when I didn't write a spiritual diary, I was hiding by myself, and there were so many sins in my heart. As I meditated on the Word of God to write a spiritual diary, I became more sensitive to sin, and quickly noticed what I had done wrong on my own without God. Now I confessed in my spiritual diary, and it became a way of repentance (Hannah, F/53).

In the past, it took a long time to repent after sin and to recover the soul. But, the spiritual recovery cycle was shortened by writing a spiritual diary every day, and writing a repentance record for the sins of the day. Afterwards, I think that when I am facing the same situation, I turn to God a little faster than before (Martha, F/48).

Participants stated that writing a spiritual diary every day helped them to experience the fullness of God and become aware of the grace received. While they dedicated their lives to pursue of fellowship with God, they experienced transformation, bringing in more of God’s holy nature and escaping from sinful habits, such as alcohol and smoking:

I think that the greatest pleasure in my life is a closer walk with God. I enjoy recording how I walk intimately with Him in my daily life and sharing this journey with church members through the website and the mobile application of http://journalwithjesus.org/. As the grace of God flow over into me and members of church group, we empower others and are empowered by them through this reciprocal sharing between church members (Jacob, M/67).

While facing difficult situations, I get angry or depressed easily, and I don't feel calm. However, when I keep a spiritual diary, I experience real changes, and no longer feel the same ups and downs (Ezra, F/53).

Several times, I failed when trying to quit drinking and smoking…. However, every time I smoked while writing my spiritual diary, I kept thinking about dying to self and living for Christ, so I quit smoking and didn't drink. In doing so, I was able to change my life completely and move into the Lord's light from the darkness (Paul, M/46).

Participants illustrated the spiritual diary as the shower of the soul and the jar containing God’s grace. Just like we wash our bodies every day in the shower, confession through daily spiritual diary writing moves what was done in the darkness into the light, where it can be healed. Moreover, it allowed them to become more conscious of God and His voice. They were able to bring their hearts to God in order to address their inner negativity in the presence of God, enabling them to move beyond their present circumstances. As a result, they were gradually transformed into God’s image and experienced the fullness of God’s presence with other church members.

In summary, the spiritual diary can be a powerful tool of spiritual discipline, leading to growth in faith and life changes. Participants reported that there was a big difference between writing and not writing spiritual diary daily. Participants who formed a godly habit of writing spiritual diaries daily could maintain a constant fellowship with God and experienced a further sanctification and transformation.

### Integrated Result

The results from the quantitative and qualitative studies provided similar information about the benefits of writing spiritual diaries in Korea. The participants who wrote spiritual diaries 5–7 times a week had significantly higher levels of spiritual growth and psychological well-being than those who wrote spiritual diaries 1–4 times a week or less than once a month. Three major themes were identified from the benefits of daily spiritual diaries reported by focus group interviewees: the acquisition of godly habits, a closer walk with Jesus, and the fullness of God’s presence. These integrated results of the mixed-method research indicate that writing a spiritual diary can be an effective religious activity leading to spiritual growth and psychological well-being.

## Discussion

The mixed-method research provided evidence of the benefits of writing spiritual diaries for improving spiritual growth and psychological well-being. Participants who wrote spiritual diaries ‘5–7 times a week’ had higher scores of spiritual perspective, self-transcendence, and satisfaction in life than those who wrote spiritual diaries less frequently. These results from quantitative data were further explored with the focus group study which provided in-depth insights into participants’ experiences with writing a spiritual diary every day. Focus group interviewees reported they experienced close fellowship with God and the fullness of God’s presence through forming godly habits of writing daily spiritual diaries.

First, the quantitative study results revealed that those who wrote spiritual diaries 5–7 times a week showed high spiritual perspective and self-transcendence scores. This indicates that the people who write spiritual diaries 5–7 times a week have a greater level of spiritual perspective and spiritual activity than those who do not (Kim et al., 2012; Reed, 1986). Also, they have greater expansions of personal boundaries within intrapersonal, interpersonal, transpersonal, and temporal domains than those who do not keep spiritual diaries (Kim et al., 2012; Reed, 1987). In other words, they have a higher awareness of the self, interest in helping others, spiritual meaning in life, and the ability to integrate time by writing spiritual diaries every day.

As reflected in the focus group interviews, we found that daily spiritual diary writing also increased their overall spiritual growth. The interviewees described how the godly habit of writing spiritual diaries on a daily basis led them to be closer to God, experiencing the presence of God more often. A spiritual diary is different from an ordinary diary: An ordinary diary records daily life, whereas a spiritual diary is training one to see Jesus inside, spending time in His company, and writing about this experience (Yoo, 2017). This training enables writers to view themselves, others, and their lives differently, thereby overcoming sin and temptation, while experiencing spiritual growth.

Furthermore, there was a significant difference in psychological well-being according to the frequency of writing spiritual diaries. Psychological well-being refers to a person's life satisfaction through integrating the emotional and cognitive aspects (Diener et al., 1985). In this study, cognitive aspects were measured by the meaning and satisfaction of life, and the emotional aspects were measured by depression. Despite no differences in depression found between the groups, there was a larger increase in life satisfaction for the daily spiritual diary group, when compared to those who wrote less frequently. There were limited direct comparisons due to a lack in prior spiritual diary research. However, a previous study examining mood changes during spiritual activities using the Daily Phone Diary for parents of children with cystic fibrosis found that such activities lowered their depression and increased their positive feelings (Szczesniak et al., 2017).

Consistent with quantitative results, the findings of the qualitative study also showed that spiritual diaries helped writers to not only look to Jesus during moments of suffering, but also to view events with increased hopefulness. Writing spiritual diaries every day allows them to reflect on their life experiences and interpret them in a more positive direction, which can help people improve overall life satisfaction.

These integrated quantitative and qualitative findings contribute to new knowledge about the benefits of spiritual diaries in regard to spiritual growth and psychological well-being. Considering various spiritual self-care activities that healthcare providers easily incorporate into patient care, a spiritual diary can be usable at anytime and anywhere without extra cost and the need for therapists.

## Limitations

The present study was conducted with a convenience sample recruited from one church in Korea. In addition, the majority of participants were mature, long-time Christians. This limits generalizing the results to other Koreans who write spiritual diaries. Also, it may be possible that spiritual diary writers from different religious and cultural backgrounds would have different responses to spiritual diaries. Further study with a larger and broader range of samples including diverse cultures and religions may be helpful. In addition, the current study could be expanded by incorporating patient and/or family samples including those with mental or physical illness.

## Conclusion

The results of this integrated quantitative and qualitative study provided evidence for the benefits of spiritual diaries in regard to spiritual growth and psychological well-being. It suggests that spiritual diary keeping could be incorporated into various spiritual disciplines, such as prayer, meditation, and worship, as a vehicle for therapeutic religious activity. These findings are particularly important for healthcare providers who want to facilitate patient self-care. Future research utilizing a quasi-experimental research design is needed to test the causal effects of spiritual diary writing on spirituality, as well as on psychological and physical well-being.

## References

[CR1] Beck AT, Ward C, Mendelson M, Mock J, Erbaugh J (1961). Beck depression inventory (BDI). Archives of General Psychiatry.

[CR2] Bengtsson M (2016). How to plan and perform a qualitative study using content analysis. NursingPlus Open.

[CR3] Cho MJ, Kim KH (1993). Diagnostic validity of the CES-D (Korean version) in the assessment of DSM-III-R major depression. Journal of Korean Neuropsychiatric Association.

[CR4] Creswell JW, Creswell JD (2017). Research design: Qualitative, quantitative, and mixed methods approaches.

[CR5] Diener ED, Emmons RA, Larsen RJ, Griffin S (1985). The satisfaction with life scale. Journal of Personality Assessment.

[CR20] Doolittle, B. R., Justice, A. C., & Fiellin, D. A. (2018). Religion, spirituality, and HIV clinical outcomes: A systematic review of the literature. *AIDS and Behavior*, *22*(6), 1792–1801. 10.1007/s10461-016-1651-z 10.1007/s10461-016-1651-z 28004218

[CR6] Kim, S. H. (2017). *Alternative consideration on the spiritual disciplines in Korean church (spiritual journal: spiritual disciplines through the spiritual journal)* (Unpublished doctoral dissertation). Wesley Theological Seminary, Jakarta.

[CR7] Kim SS, Cheon YK, Choi EJ, Gil MJ (2018). Spiritual diary, spiritual growth, psychological and physical well–being. Korean Journal of Christian Counseling.

[CR8] Kim SS, Reed PG, Kang Y, Oh J (2012). Translation and psychometric testing of the Korean versions of the spiritual perspective scale and the self-transcendence scale in Korean elders. Journal of Korean Academy of Nursing.

[CR9] Kim WS, Kim YJ (1997). The relationship between subjective well-being and social activity. The Korean Psychological Association: Culture and Social Issues.

[CR10] Park N (2010). A study on the effect of spiritual diary as a spiritual training. Theology and Context.

[CR21] Park, C. L., & Slattery, J. M. (2013). Religion, spirituality, and mental health. In R. F. Paloutzian & C. L. Park (Eds.), *Handbook of the psychology of religion and spirituality* (pp. 540–559). The Guilford Press.

[CR11] Radloff LS (1977). The CES-D scale: A self-report depression scale for research in the general population. Applied Psychological Measurement.

[CR12] Reed PG (1986). Developmental resources and depression in the elderly. Nursing Research.

[CR13] Reed PG (1987). Spirituality and well-being in terminally ill hospitalized adults. Research in Nursing and Health.

[CR22] Salsman, J. M., Pustejovsky, J. E., Jim, H. S. L., Munoz, A. R., Merluzzi, T. V., George, L., Park, C. L., Danhauer, S. C., Sherman, A. C., Snyder, M. A., & Fitchett, G. (2015). A meta-analytic approach to examining the correlation between religion/spirituality and mental health in cancer. *Cancer*, *121*(21), 3769–3778. 10.1002/cncr.29350 10.1002/cncr.29350 PMC4618157 26258536

[CR14] Schnitker SA, Richardson KL (2019). Framing gratitude journaling as prayer amplifies its hedonic and eudaimonic well-being, but not health, benefits. The Journal of Positive Psychology.

[CR23] Simão, T. P., Caldeira, S., & De Carvalho, E. C. (2016). The effect of prayer on patients’ health: Systematic literature review. *Religions*, *7*(1), 1–11. 10.3390/rel7010011

[CR15] Szczesniak RD, Zou Y, Dimitriou SM, Quittner AL, Grossoehme DH (2017). Use of the daily phone diary to study religiosity and mood: Convergent validity. Journal of Health Care Chaplaincy.

[CR16] Wesley, J. (1996). The complete works of John Wesley, Volume 11, thought, addresses, prayers, letters [The Ages Digital Library version]. Retrieved from https://ru.b-ok2.org/ireader/3195217

[CR24] White, M. L. (2016). Spirituality self-care practices as a mediator between quality of life and depression. *Religions*, *7*(5), 54. 10.3390/rel7050054

[CR17] With Jesus Ministry [Website]. (2021, Jan 20). Retrieved from http://wjsm.kr/

[CR18] Yoo K (2017). Spiritual Journal.

[CR19] Yoo, K., & Noh, P. S. (2018). The Korean Cyber Monastery Movement. *Lausanne global analysis, 7*(5). Retrieved from https://www.lausanne.org/content/lga/2018-09/the-korean-cyber-monastery-movement

